# Multi-layered metabolic effects of trehalose on the liver proteome in apoE-knockout mice model of liver steatosis

**DOI:** 10.1007/s43440-024-00615-3

**Published:** 2024-06-24

**Authors:** Weronika Pogoda, Jakub Koczur, Aneta Stachowicz, Józef Madej, Rafał Olszanecki, Maciej Suski

**Affiliations:** 1 Proteomics Laboratory, Centre for the Development of Therapies for Civilization and Age-Related Diseases, Jagiellonian University Medical College, Krakow, Poland; 2 Department of Pharmacology, Faculty of Medicine, Jagiellonian University Medical College, Kraków, Poland

**Keywords:** Trehalose, Apoe-knockout mice, Liver steatosis, Beta-oxidation, Proteomics

## Abstract

**Background:**

Metabolic dysfunction-associated fatty liver disease has been well documented as a key independent risk factor for the development of atherosclerosis. A growing body of evidence suggests that due to its numerous favorable molecular effects, trehalose may exert beneficial effects in counteracting liver steatosis. In our previous study, we described the antiatherosclerotic and antisteatotic properties of trehalose, which we attributed to the induction of autophagy. Considering the pleiotropic activities of trehalose, our present study aimed to extend our preliminary results with the comprehensive examination of proteome-wide changes in the livers of high-fat-fed apoE-/- mice.

**Methods:**

Thus, we applied modern, next-generation proteomic methodology to comprehensively analyze the effects of trehalose on the alterations of liver proteins in apoE-/- mice.

**Results:**

Our proteomic analysis showed that the administration of trehalose elicited profound changes in the liver proteome of apoE-/- mice. The collected data allowed the identification and quantitation of 3 681 protein groups of which 129 were significantly regulated in the livers of trehalose-treated apoE-/- mice.

**Conclusions:**

The presented results are the first to highlight the effects of disaccharide on the induction of proteins mainly related to the metabolism and elimination of lipids, especially by peroxisomal β-oxidation. Our study provides evidence for the pleiotropic activity of trehalose, extending our initial observations of its potential mechanisms responsible for mitigating of liver steatosis, which paves the way for new pharmacological strategies in fatty liver disease.

**Supplementary Information:**

The online version contains supplementary material available at 10.1007/s43440-024-00615-3.

## Introduction

Despite the clinical and research efforts over decades, cardiovascular diseases (CVD) remain a rapidly growing health problem in Western countries [1]. Atherosclerosis results in life-threatening occlusion of the blood vessels as a consequence of lipid-driven plaque buildup, characterized by endothelial activation, accumulation of oxidized low-density lipoproteins, and subsequent migration of monocytes and other inflammatory cells [2]. Importantly, it has been well documented that metabolic dysfunction-associated fatty liver disease (MAFLD) is a key independent risk factor for the development of atherosclerosis [3] as abnormal liver lipid metabolism accompanies atherosclerogenesis through mutually overlapping and amplifying parallel mechanisms [4]. The etiology of MAFLD encompasses numerous factors including dyslipidemia, obesity, dysregulation of fat tissue metabolism, insulin resistance, oxidative stress, mitochondrial dysfunction, systemic inflammation, and altered intestinal microbiota composition [5–7]. At the molecular level, MAFLD manifests itself in increased uptake of circulating lipids, increased lipogenesis, and disturbed lipid utilization through β-oxidation [7, 8].

Trehalose is a naturally occurring disaccharide composed of two glucose molecules connected by an α-1,1 linkage, which plays a vital role not only as an energy source but also as a protective agent against various types of environmental stress, like heat, cold, dehydration, or oxidation [9]. It also has an interesting property as a direct protein stabilizer and antiaggregatory agent [10]. Furthermore, a growing body of evidence suggests that trehalose, i.a. by regulating AMPK and NRF2 signaling, has numerous beneficial molecular effects, including controlling glucose homeostasis, attenuation of adipocyte hypertrophy, reduction of oxidative stress, regulation of insulin response in an autophagy-dependent and autophagy-independent manner [11–14].

In our previous study, we described the antiatherosclerotic and antisteatotic properties of trehalose [15]. The apolipoprotein E-knockout mice (apoE-/-) fed standard chow or a high-fat diet exhibited a profound formation of atherosclerotic plaques, but only the animals on a high-fat diet exhibited macrovesicular steatosis changes in the liver. Interestingly, trehalose administration led to the amelioration of atherosclerosis development only in mice fed with chow but not a high-fat diet [15]. On the contrary, the administered disaccharide exhibited beneficial effects in the livers of high fat-fed apoE -/- mice, including the reduction of macrovesicular steatosis, triglyceride deposition, and plasma content as well as the level of alanine aminotransferase (ALT). Finally, we attributed the beneficial action of trehalose in the liver to the induction of autophagy.

Considering the growing body of evidence of additional pleiotropic activities of trehalose, we aimed to extend our preliminary results with the investigation of the comprehensive proteome-wide changes in the livers of high-fat-fed apoE-/- mice upon treatment with orally administered trehalose. We hypothesized that by applying modern, next-generation proteomic (NGP) methodology we can identify additional auxiliary mechanisms beyond induction of autophagy that will unravel the complex trehalose activity in MAFLD.

## Materials and methods

### Animal experiment

The animal experiment was described in detail in [15]. Briefly, fourteen female apoE–knockout mice (apoE-/-) mice on C57BL/6J background were purchased from Taconic (Ejby, Denmark). The animals were kept in 12 h dark/12 h light cycles in air-conditioned rooms (22.5 ± 0.5 °C, 50 ± 5% humidity) with access to water ad libitum and diet. The mice were put on a high-fat (HFD) diet made by Morawski (Kcynia, Poland) at the age of 8 weeks for 16 weeks. The animals were randomly divided into two experimental groups: apoE-/- mice on a high-fat diet (*n* = 7), and female apoE-/- mice on a high-fat diet treated with trehalose (*n* = 7). Trehalose (α-D-Glucopyranosyl-α-D-glucopyranoside, Sigma-Aldrich, Saint-Louis, MO, PN: T0167) was mixed without heating with the diet at a dose of 2.5 g per kg of body weight per day. The animals were injected with 1000 IU of fraxiparine i.p (Sanofi-Synthelabo, Paris, France) at the age of 6 months and euthanized in a chamber filled with carbon dioxide. The livers were then dissected and stored at -80 °C for proteomic analysis. All animal procedures were performed according to the guidelines from Directive 2010/63/EU of the European Parliament on the protection of animals used for scientific purposes and approved by the Jagiellonian University Ethics Committee on Animal Experiments (no. 73/2011).

### Sample preparation, LC-MS/MS measurements and data analysis

The mass spectrometry-based label-free quantitative proteome analysis is described in detail in [16]. Briefly, the liver samples were lysed in 2% SDS, 50 mM DTT in 0.1 M Tris–HCl pH 7.6. After protein concentration determination [17] a volume containing 70 µg of total protein was digested to peptides using the filter-aided sample preparation (FASP) protocol [18]. Project-specific spectral library was performed by HpH fractionation, while all analyzed samples were included in the library pool to comprehensively represent the study groups.

Peptides (1 µg) were separated on reversed-phased C18 column operating in nanoflow regime and applied to a TripleTOF 6600+ (Sciex, Framingham, MA) mass spectrometer operating in DDA acquisition mode for library preparation and in the SWATH acquisition mode for quantitative proteomic analysis, as described in detail in [16].

DDA data were searched against the mouse UniProt database (release 2021_01_04, 17,056 entries) and the MaxQuant Contaminants list (245 entries) using the Pulsar search engine in Spectronaut 18 software (Biognosys, Schlieren, Switzerland) [19] with default parameters. Created spectral library was used as a template for SWATH data analysis. Data were filtered by 1% FDR at the peptide and protein level, while quantitation and interference correction were performed at the MS2 level. Protein grouping was performed by ID picker algorithm [20]. Data normalization was performed by means of a global regression strategy, while differential protein abundance was validated using Student’s t-tests with multiple testing corrections after Storey [21]. The LC-MS data, library, and the Spectronaut project have been deposited to the ProteomeXchange Consortium via the PRIDE partner repository [22] with the dataset identifier PXD049421.

### Pathway annotation and functional grouping

Functional grouping and pathway annotations were performed using ClueGO [23] in the Cytoscape 3.8.2 software environment [24]. CORUM3.0 (release 03.09.2018), KEGG (release 17.02.2020), REACTOME (release 17.02.2020), and WikiPathways (release 17.02.2020) pathways as well as all GO ontologies were used in the analysis. The enrichment results were validated by a two-sided enrichment/depletion geometric statistical test with Bonferroni step down as a p-value correction method. The minimum and maximum GO levels were set as 1 and 4, respectively, with the cluster criteria of a minimum of 3 genes that comprise a minimum of 2% of the GO term. The kappa score threshold was set as 0.4.

### Western blot

Liver lysates were mixed with gel loading buffer (50 mM Tris, 10% SDS, 10% glycerol, 2 mg/ml bromophenol blue) in a 4:1 ratio (v/v) and incubated at 96 °C for 5 min. Samples (10 µg of protein per well) were separated on SDS-polyacrylamide gels (12%) (Mini Protean II, Bio-Rad, Hercules, CA) using a Laemmli buffer system and semi-dry transferred to nitrocellulose membranes (GE Healthcare, Chicago, IL). The membranes were blocked with 5% bovine serum albumin in TTBS at room temperature for 1 h and incubated overnight at 4 °C temperature with a specific primary antibody for 1:1000 CSAD (MyBioSource, San Diego, CA, PN: MBS9133861). Incubation with secondary HRP-conjugated antibodies (GE Healthcare, Chicago, IL, PN: NA934V) was performed at room temperature for 1 h (dilution 1:5000). Bands were developed with the use of ECL Prime reagents (GE Healthcare, Chicago, IL). Kaleidoscope markers (BioRad, Hercules, CA, PN: 161–0395) were used for molecular weight determinations. Normalization of protein band intensities was performed using a total protein measurement approach. Protein pattern images were taken using an ImageQuant LAS 500 scanner (GE Healthcare, Chicago, IL).

### Taurine ELISA

The taurine assay kit (Cell Biolabs, San Diego, CA, PN: MET-5071) was used according to the manufacturer’s guidelines. The taurine level was normalized to the total protein concentration.

### Statistics

All quantitative data from molecular biology methods were log2-transformed before statistical testing. The equality of variances was evaluated using the F test (two-group comparisons), while the normality of the data and their residuals was evaluated using the Shapiro–Wilk test. The Student’s t-test was used to assign the statistical significance of the Western blot and ELISA data in the GraphPad Prism 10.1.2 software (GraphPad Prism, San Diego, CA). *p* < 0.05 was considered statistically significant.

## Results

**Fig. 1:**
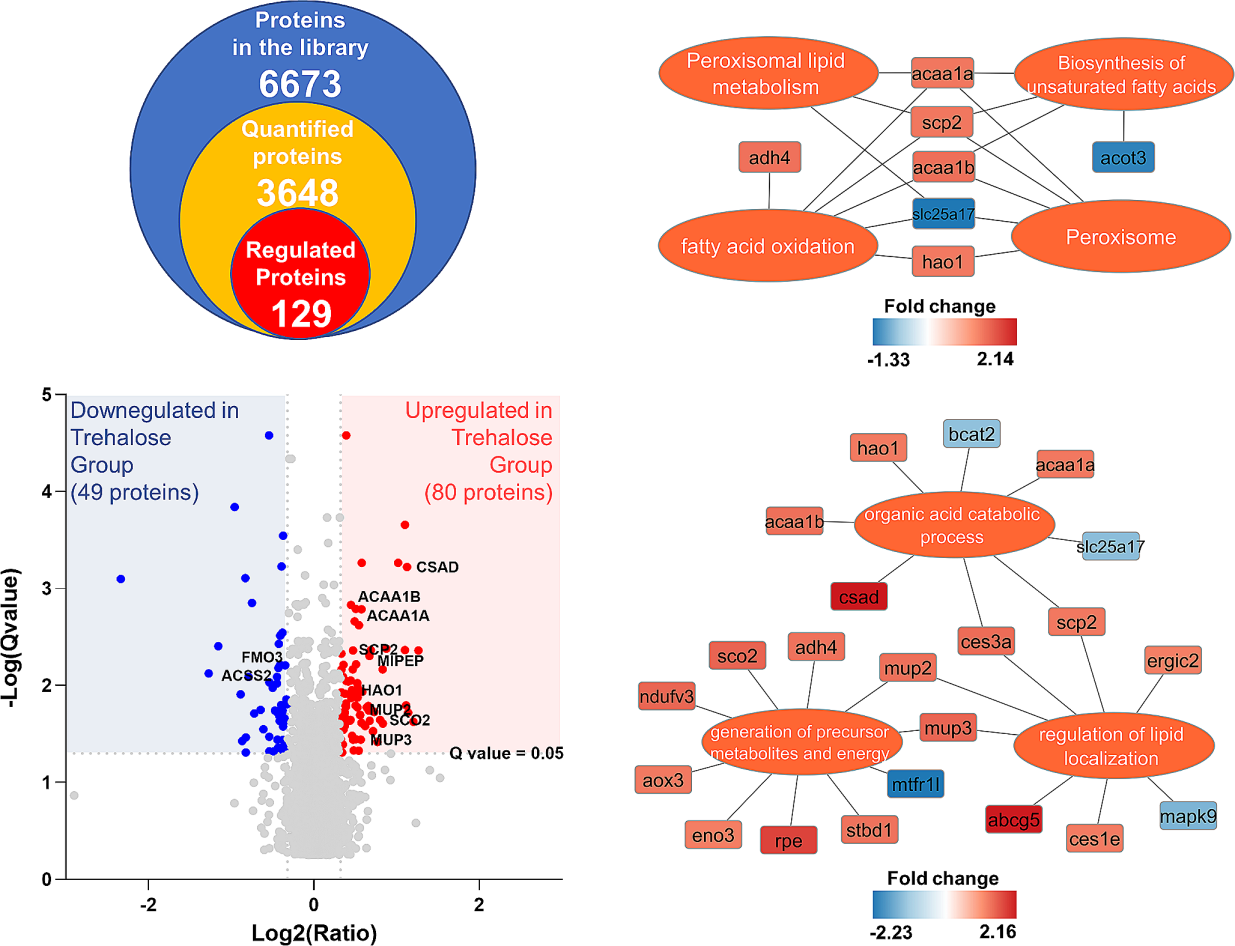
**Functional grouping and pathway annotations of the differentially regulated proteins in trehalose-treated apoE-/- mice livers.** Ontology and pathway enrichment point to the key processes regulated by trehalose, including peroxisomal β-oxidation as the main feature. The enrichment results were validated by a two-sided enrichment/depletion geometric statistical test with Bonferroni step down as a p-value correction method

**Fig. 2:**
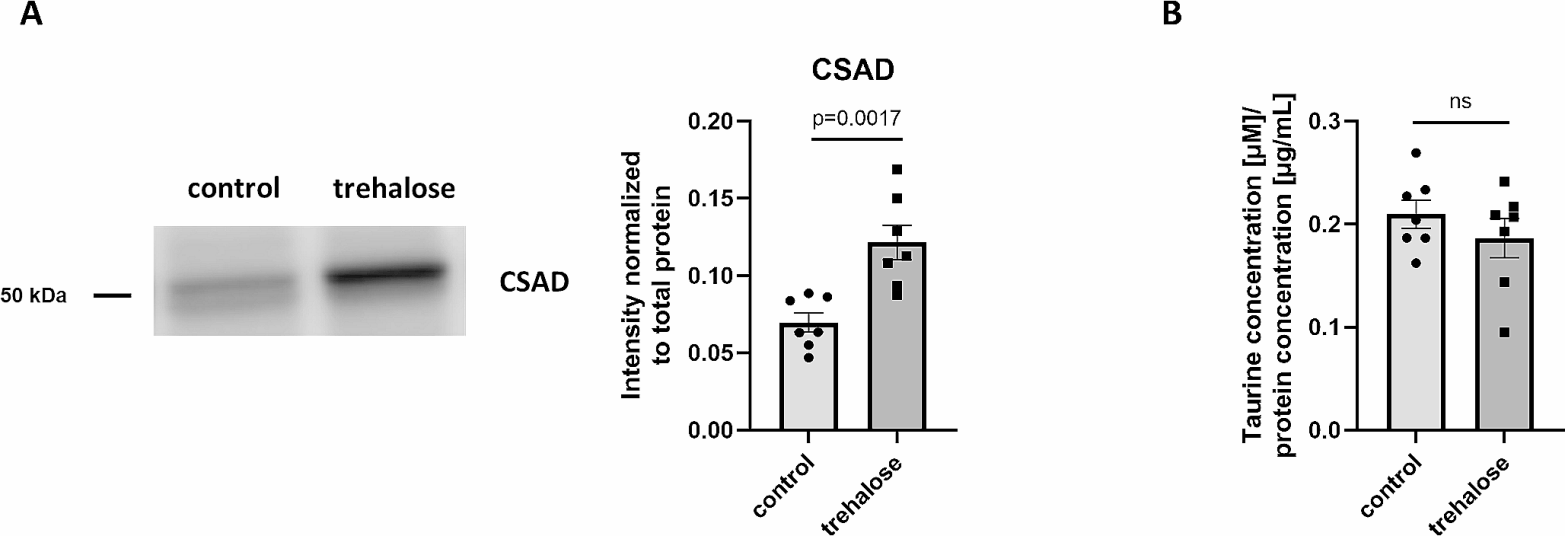
**Validation of cysteine sulfinic acid decarboxylase (CSAD) expression and activity** Western blot measurement confirmed the trehalose-elicited induction of CSAD (A) which was not accompanied by an elevation of taurine in the liver (B). Data are represented as mean ± SEM, *n* = 7 (*p* = 0.0017, t = 4.029, df = 12). Data were validated by an unpaired Student’s t-test, with the F test used to evaluate the equality of variances and Shapiro–Wilk test to verify the normality of the data and their residuals

The acquired SWATH data were analyzed using Spectronaut with default settings. Median protein group CVs were calculated for 10.91% and 10.59% for the control and trehalose-treated groups, respectively, which allowed for the estimation of a significant quantitative cutoff for an absolute 1.25-fold change (statistical power 99.2%). On average, collected data allowed for the identification and quantitation of 3 681 protein groups of which 129 were significantly regulated in livers of trehalose-treated apoE-/- mice (Suppl. Tab. S1): 80 were upregulated, while 49 were repressed (Suppl. Fig. S1). As expected, many metabolic enzymes were regulated, including those involved in fatty acid oxidation (Fig. 1). Interestingly, we identified peroxisomes as the main cellular compartment in which trehalose-elicited metabolic alterations were present (Fig. 1, Suppl. Tab. S1). We identified several quantitative differences in the abundances of proteins that form a functional network cluster related to fatty acid metabolism in peroxisomes, with 3-ketoacyl-CoA thiolase A and B (ACAA1 A/B) and sterol carrier protein 2 (SCP2) as the main characteristics. These changes were accompanied by the induction of cysteine sulfinic acid decarboxylase (CSAD) and the major urinary protein isoforms (MUP2 and MUP3) as well as mitochondrial intermediate peptidase (MIPEP) and cytochrome c oxidase 2 (SCO2), which collectively indicate a possible involvement of mitochondria in trehalose-elicited changes in the livers of apoE-/- mice, however, the electron transport chain proteins and mitochondrial-specific β-oxidation enzymes were not altered. Moreover, we validated the expression of CSAD and its activity by targeted molecular methods and found that trehalose-elicited induction of CSAD abundance (fold change 1.74, *p* = 0.0017) was not accompanied by the increase in the level of liver taurine (Fig. 2, Suppl. Fig. S1).

## Discussion

In the present study we have examined the effects of prolonged administration of trehalose on the liver proteome of apolipoprotein E knockout (apoE-/-) mice on a high-fat diet (HFD). We aimed to explore the novel potential mechanisms by which this disaccharide ameliorates MAFLD. Previously [15] we identified autophagy as a possible driving feature of trehalose activity. Here, we provide additional indirect evidence of the possible regulation of autophagy elicited by trehalose (for example, induction of the endoplasmic reticulum-Golgi intermediate compartment protein 2, ERGIC2). However, the main finding of our present quantitative proteomic measurements is that trehalose administration led to substantial changes in proteins related to lipid metabolism and transport and pointed to peroxisomes as a possible site of increased lipid degradation (Fig. 1). Additionally, among the 129 identified regulated proteins, we found several novel discoveries that were not previously associated with trehalose biological activities (Fig. 1, Suppl. Tab. S1), including major urinary proteins and other cellular metabolic regulators that evidence pleiotropic trehalose activity in the context of MAFLD.

Impaired fatty acid oxidation is one of the key hallmarks of MAFLD, with excessive lipid uptake and enhanced SREBP1c-mediated *de novo* lipogenesis as two simultaneous drivers of liver steatosis [25]. Our quantitative proteomic data evidenced that the amelioration of lipid deposition by trehalose treatment might result from its enhanced utilization through β-oxidation in peroxisomes (Fig. 1) with simultaneous inhibition of lipogenesis. Among others, we observed an increase in the expression of ACAA1 A/B and SCP2 proteins in trehalose-treated apoE-/- mice livers (Fig. 1). ACAA1 A/B are responsible for the thiolytic cleavage of straight-chain 3-keto fatty acyl-CoAs (3-oxoacyl-CoAs) [26, 27], while SCP2 plays a crucial role in the peroxisomal oxidation of branched-chain fatty acids [28]. Studies using peroxisomal fatty acyl-CoA oxidase knockout mice (ACOX-/-) highlight the pivotal role of ACAA1B repression in the decline of peroxisomal β-oxidation [26, 29]. Additionally, ACAA1B limits the cholesterol content in the liver by modulating the expression of enzymes involved in its biosynthesis [27]. Similarly, SCP2 is one of the most abundant peroxisomal proteins [8], while its deficiency is directly related to hepatic lipid accumulation [31]. Importantly, SCP2 can improve fatty liver by inhibiting fatty acid biosynthesis, its increased utilization through increased mitochondrial fatty acid oxidation, and by enhanced esterification and excretion of cholesterol [30, 31]. Importantly, we have simultaneously identified the decrease in the expression of acetyl-CoA synthetase (ACSS2), which is the direct target of sterol regulatory element-binding proteins (SREBPs) in lipogenesis pathways [32] and over two-fold induction of the ATP-binding cassette sub-family G member 5 (ABCG5) responsible for the bile excretion of sterols [33, 34] (Fig. 1). Taken together, our proteomic data strongly point to peroxisomes as an important site of trehalose activity in the liver of the apoE-/- mice model, which may be responsible for the amelioration of steatosis.

Moreover, we have identified several other proteins functionally involved in the metabolism of lipids that were affected by trehalose administration in apoE-/- mice livers, which can be interpreted as additional regulators mitigating the hepatic steatosis (Fig. 1). For instance, we detected over two-fold increase in CSAD (Fig. 1, Suppl. Tab. S1), which was not accompanied by an elevation of taurine in the liver (Fig. 2). This observation is consistent with that of Tan et al., who recently demonstrated that CSAD decreases significantly in patients with MAFLD and animal models, while its overexpression alleviates fatty liver progression [35]. CSAD induction was associated with the upregulation of genes related to fatty acid β-oxidation independently of the taurine pathway. These data reinforce the notion arising from our proteomic analysis that trehalose may play a dual role in the amelioration of liver steatosis through inhibition of lipid biogenesis and its enhanced degradation in peroxisomes; however, the precise molecular mechanisms of such activities remain to be established. Next, trehalose administration led to the induction of major urinary proteins in apoE-/- mice livers (Fig. 1). MUPs were shown to be involved in the regulation of lipid and glucose metabolism in skeletal muscle and liver [36, 37], while their knockout drives lipid accumulation in the liver and blood [38]. Interestingly, we have previously identified that MUPs were upregulated in liver mitochondria of apoE -/- mice compared to their wild-type counterparts [39], while their abundance decreased in mitochondria isolated from the liver of double (apolipoprotein E and eNOS) knockout mice (apoE / eNOS-DKO) compared to the apoE-/- group [40], proving their involvement in hepatic lipid homeostasis. In the present study, we have identified two MUP isoforms (MUP2 and MUP3) induced after trehalose administration (Fig. 1, Suppl. Tab. S1). Thus, we are tempted to speculate that by MUPs induction trehalose can reinforce both lipid utilization and elimination in apoE-/- mice. Furthermore, trehalose administration led to the induction of mitochondrial intermediate peptidase (MIPEP) and synthesis of cytochrome c oxidase 2 (SCO2), proteins primarily involved in the maturation of oxidative phosphorylation (OXPHOS)-related proteins [41] and cytochrome c oxidase subunit II [42], respectively. Finally, we identified the induction of iron-sulfur cluster assembly enzyme (ISCU) in trehalose-treated apoE-/- mice (Suppl. Tab. S1), a scaffold protein of the core iron-sulfur cluster (ISC) assembly complex, which provides the structural architecture on which the [2Fe-2 S] clusters are assembled [43]. The deficit in the Fe-S clusters leads to the accumulation of fatty acids because iron-dependent enzymes cannot function effectively and therefore efficient fatty acid oxidation does not occur [44]. Instead, increased hepatic iron concentration contributes to the progression of MAFLD mainly through exacerbation of oxidative stress [45].

In conclusion, our proteomic analysis showed that trehalose administration elicited profound changes in the liver proteome of apoE-/- mice. The presented results are the first to highlight the effects of disaccharide in the induction of proteins related mainly to lipid metabolism and elimination, particularly to peroxisomal β-oxidation as the main feature. Our study provides evidence for the pleiotropic activity of trehalose, which expands our initial observations regarding autophagy as one of the potential mechanisms responsible for the mitigation of MAFLD. Presented results can be used as a blueprint for novel pharmacotherapy directions for fatty liver disease and new drugs developed for more effective, multi-layered action.

### Electronic supplementary material

Below is the link to the electronic supplementary material.

Supplementary material 1

Supplementary material 2

## Data Availability

The dataset generated during and analyzed during the current study are available from the corresponding author on reasonable request.

## Abbreviations

ABCG5: ATP-binding cassette sub-family G member 5
ACAA1 A/B − 3: ketoacyl-CoA thiolase A / B
ACN: acetonitrile
ACOX: acyl-CoA oxidase
ACSS: acetyl-CoA synthetase
ALT: alanine aminotransferase
AMPK − 5'AMP: activated protein kinase
apoE: /--apolipoprotein E-knockout mice
ATP: adenosine triphosphate
CID: collision-induced dissociation (in MS/MS scans)
CSAD: cysteine sulfinic acid decarboxylase
CUR: curtain gas
CVDs: cardiovascular diseases
CVs: coefficients of variation
DDA: data-dependent acquisition
DKO: double knockout
DTT: dithiothreitol
ELISA: enzyme-linked immunosorbent assay
eNOS: endothelial nitric oxide synthase
ERGIC2: endoplasmic reticulum-Golgi intermediate compartment protein 2
FA: formic acid
FASP: filter-aided sample preparation
FDR: false discovery rate
GS1: ion source gas 1
HAO1: hydroxyacid oxidase
HFD: high-fat diet
HpH: high-pH fractionation
HRP antibodies: horseradish peroxidase secondary antibodies
IHT: interface heater temperature
i.p: intraperitoneal (injection)
iRT: indexed Retention Time (peptide mix)
ISC: iron-sulfur cluster
ISCU: iron-sulfur cluster assembly enzyme
kDa: kilodaltons (molecular weight)
LC: MS/MS-liquid chromatography-tandem mass spectrometry
LysC: lysyl endopeptidase® (digestive enzyme)
MIPEP: mitochondrial intermediate peptidase
MUP(s): major urinary protein(s)
MAFLD: metabolic dysfunction-associated fatty liver disease
NGP: next-generation proteomic
NRF2: nuclear factor erythroid 2-related factor 2
OXPHOS: oxidative phosphorylation
PPARα: peroxisome proliferator-activated receptor
PSM: peptide-spectrum match
ROS: reactive oxygen species
SCO2: cytochrome c oxidase 2
SCP2: sterol carrier protein 2
SDS: sodium dodecyl sulfate
SREBPs: sterol regulatory element-binding proteins
SWATH: sequential windowed acquisition of all theoretical fragments
TMA: trimethylamine
TMAO: trimethylamine N-oxide
WF: tryptophan fluorescence
