# Supplementary figures and images for: Multi-layered metabolic effects of trehalose on the liver proteome in apoE-knockout mice model of liver steatosis
Source: Pharmacol Rep. 2024 Jun 24;76(4):902–9. doi: 10.1007/s43440-024-00615-3 (PMC11294376; doi:10.1007/s43440-024-00615-3)

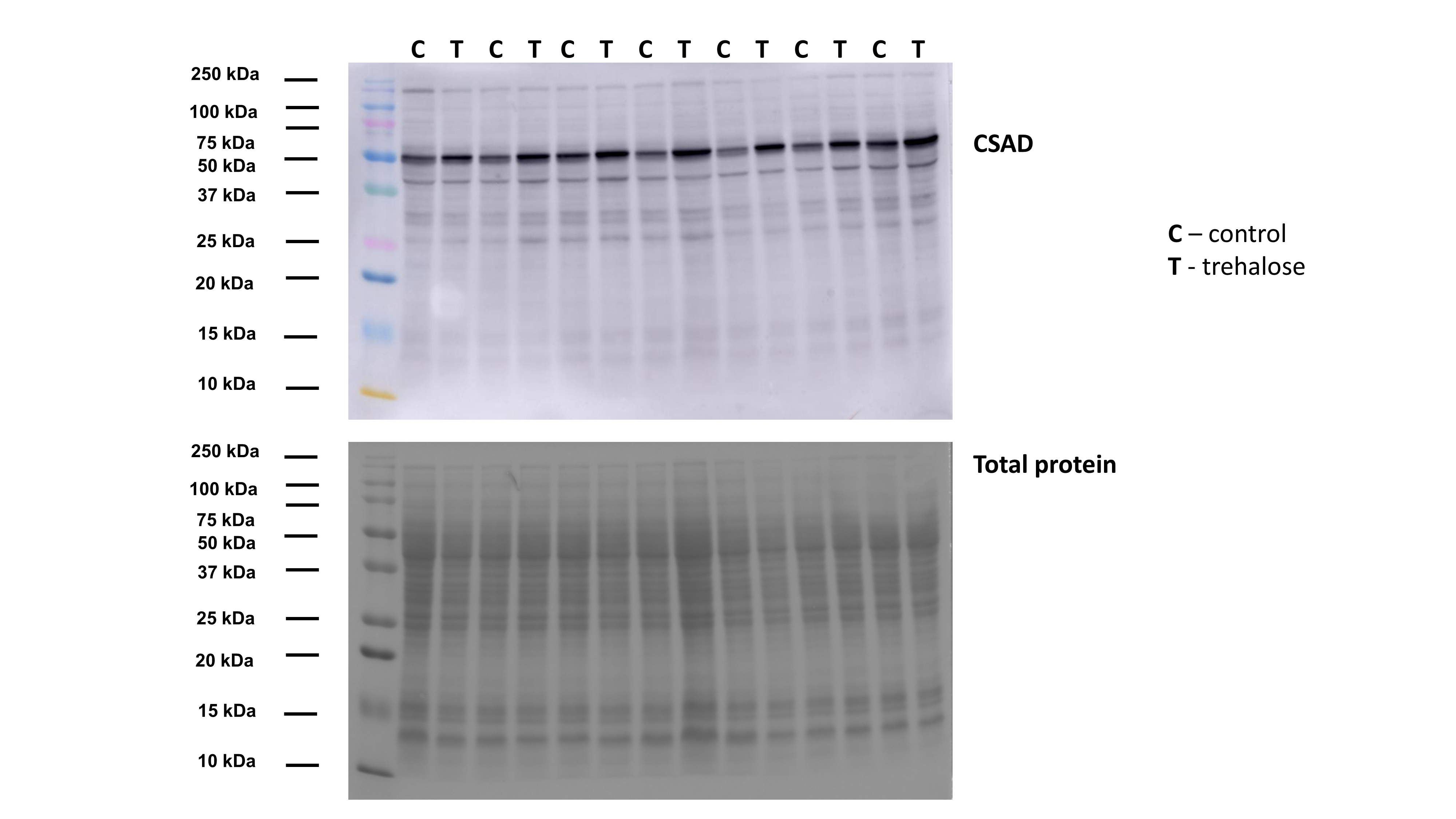

Supplement: Supplementary file 2 — Supplementary material 2 [file 43440_2024_615_MOESM2_ESM.tif]
